# Clinical implementation of respiratory‐gated spot‐scanning proton therapy: An efficiency analysis of active motion management

**DOI:** 10.1002/acm2.12584

**Published:** 2019-04-10

**Authors:** Edgar Gelover, Amanda J. Deisher, Michael G. Herman, Jedediah E. Johnson, Jon J. Kruse, Erik J. Tryggestad

**Affiliations:** ^1^ Department of Radiation Oncology Mayo Clinic Rochester Rochester MN USA

**Keywords:** breath‐holding, motion management, phase‐gating, proton therapy, spot‐scanning

## Abstract

**Purpose:**

The aim of this work is to describe the clinical implementation of respiratory‐gated spot‐scanning proton therapy (SSPT) for the treatment of thoracic and abdominal moving targets. The experience of our institution is summarized, from initial acceptance and commissioning tests to the development of standard clinical operating procedures for simulation, motion assessment, motion mitigation, treatment planning, and gated SSPT treatment delivery.

**Materials and methods:**

A custom respiratory gating interface incorporating the Real‐Time Position Management System (RPM, Varian Medical Systems, Inc., Palo Alto, CA, USA) was developed in‐house for our synchrotron‐based delivery system. To assess gating performance, a motion phantom and radiochromic films were used to compare gated vs nongated delivery. Site‐specific treatment planning protocols and conservative motion cutoffs were developed, allowing for free‐breathing (FB), breath‐holding (BH), or phase‐gating (Ph‐G). Room usage efficiency of BH and Ph‐G treatments was retrospectively evaluated using beam delivery data retrieved from our record and verify system and DICOM files from patient‐specific quality assurance (QA) procedures.

**Results:**

More than 70 patients were treated using active motion management between the launch of our motion mitigation program in October 2015 and the end date of data collection of this study in January 2018. During acceptance procedures, we found that overall system latency is clinically‐suitable for Ph‐G. Regarding room usage efficiency, the average number of energy layers delivered per minute was <10 for Ph‐G, 10‐15 for BH and ≥15 for FB, making Ph‐G the slowest treatment modality. When comparing to continuous delivery measured during pretreatment QA procedures, the median values of BH treatment time were extended from 6.6 to 9.3 min (+48%). Ph‐G treatments were extended from 7.3 to 13.0 min (+82%).

**Conclusions:**

Active motion management has been crucial to the overall success of our SSPT program. Nevertheless, our conservative approach has come with an efficiency cost that is more noticeable in Ph‐G treatments and should be considered in decision‐making.

## 1 INTRODUCTION

A four‐dimensional computed tomography (4D‐CT) study obtained at time of treatment simulation, used to approximate motion throughout treatment, is the current gold standard for motion assessment for spot‐scanning proton therapy (SSPT). However, using the 4D‐CT to define a geometric uncertainty margin for targets and organ at risks (OARs) is typically not adequate for SSPT given that (a) protons are extremely sensitive to heterogeneities in their path^1^ (i.e., their water‐equivalent depth, WET)^2^ and (b) tumor motion in the context of an energetically‐ and spatially sequential SSPT delivery can result in dosimetric patterns of constructive and destructive interference — so called “motion interplay”.^3^, ^4^, ^5^

WET variation has the potential to produce a significantly different dose distribution for any defined sub‐portion of the respiratory cycle. Without motion management, movement of both the target and upstream normal tissues along the beam path (such as the diaphragm) can lead to unacceptable differences in planned vs delivered proton range.^6^ The severity of the effect is beam specific. Motion interplay in SSPT can occur both as a consequence of target motion (perpendicular to beam direction) and also as a consequence of WET changes along the beam path associated with motion — that is, volumetric interplay.^7^ The overall magnitude of the associated dose perturbation depends on the target motion dynamics, spot delivery/timing parameters, and their mutual degree of synchronization. A well‐demonstrated mitigation technique for the interplay effect is “repainting,” also referred to as rescanning. Depending on the capabilities of the delivery system, the associated spot rescanning pattern can be delivered in a volumetric mode (i.e., delivering the whole field N times in succession with a spot weight reduction per volume rescan of 1/N) or each layer can be fully or partially rescanned in succession, the latter having been developed with numerous variants such as iso‐layered rescanning,^8^, ^9^ scaled rescanning,^8^ and the recently developed evenly spread spot‐adapted rescanning.^10^ An alternative basic/simple mitigation strategy for the interplay effect that has been shown to be effective is the use of larger pristine proton spots,^3^ for example, through use of a range shifter if available. Other related motion management strategies that can mitigate interplay include: aligning the preferential directionality of spot scanning with the principle (cranial‐caudal) motion direction; the use of fractionation^11^; and reduction of target motion through actively gated beam delivery or some form of mechanical restrictions such as compression.^12^, ^13^ Lastly, not all SSPT optimization approaches are equivalent in terms of motion robustness. Two adopted frameworks are single‐field optimization (SFO) and multi‐field optimization (MFO). Per‐field dose distributions derived from Multi‐field Optimization (MFO) tend to be more heterogeneous than SFO fields and are, therefore, more sensitive to anatomical variation, inclusive of motion‐related variation.

For these reasons, target motion in the context of SSPT requires a mitigation strategy. At our institution, target motion is managed with a potential combination of techniques, chosen on the basis of target motion amplitude and treatment site. We present a summary of our experience combining motion‐mitigation and SSPT. The PTCOG Thoracic and Lymphoma Subcommittee published initial guidelines^14^ on implementing active motion management, the launch of our program, however, precedes the release date of that report, in addition, this manuscript presents an efficiency study on patient data.

## 2 MATERIALS AND METHODS

### 2.A. System components

#### 2.A.1. Proton facility and accelerator system

A description of our proton center can be found in Table 1. Our Hitachi synchrotron‐based system (Hitachi Americas, Ltd.) is capable of producing 97 separate proton energies between 71.3 and 228.8 MeV; range shifters of 25 and 45 mm WET can be incorporated for delivering spots superficially. The pristine Bragg peak width in water ranges from 1.2 mm at 71.3 MeV to 8.3 mm at 228.8 MeV (R80_distal_–R80_proximal_). The spot size (*σ*) near isocenter/end‐of‐range in water varies between approximately 4 and 10 mm, depending on incident beam energy and optional choice of range shifter. In a previous publication,^15^ the following relevant beam parameters were determined experimentally: average magnet preparation and verification time: 1.93 ms, average scanning speeds: 5.9 and 19.3 m/s in x and y directions, respectively, and the maximum proton charge available for one acceleration is 2.0 ± 0.4 nC.

**Table 1:** Technical description of proton facility

Accelerator type	Synchrotron
Manufacturer	Hitachi (Probeat V5; Hitachi Americas, Ltd.)
Depth scanning energies	71.3–228.8 MeV
Delivery technique	Discrete spot scanning
Number of isocentric gantries	4
Gantry rotation	Half‐gantries (190°)
Number of fixed‐beam research rooms	1
Field size	30 cm × 40 cm
Average switch layer time	~2 s
2 Gy delivery time (10×10×10 cm^3^ SOBP)	~120 s
Minimum MU limit	0.002
TPS for robust planning	Eclipse (v15.1; Varian Medical Systems, Inc.)
Data management (R&V System)	ARIA (v15.1; Varian Medical Systems, Inc.)
Operation starting date	June 2015

Monitor Units (MUs) were defined during beam commissioning adapting the methodology described by Gillin et al.^16^ Briefly, an appropriately uniform one liter dose distribution (10 × 10 × 10 cm^3^), comprised of 10 115 discrete spots and delivered on a 6 mm spatial grid including 27 energy layers between 121.0 and 173.6 MeV with nominal mid‐SOBP depth of 15 cm, was delivered to a water tank with gantry at 0°. We calibrated charge (or vendor‐defined pulses) per MU in each of the two monitor chambers by arbitrarily asserting that the total required MU (202.82) to define the calibration SOPB must result a dose measurement of 202.82 cGy at the mid‐SOBP depth. In determining dose we followed the established protocol from the International Atomic Energy Agency (IAEA) TRS 398^17^ using a calibrated Farmer chamber (PTW N30013).

#### 2.A.2. Gating interface

**Figure 1:**
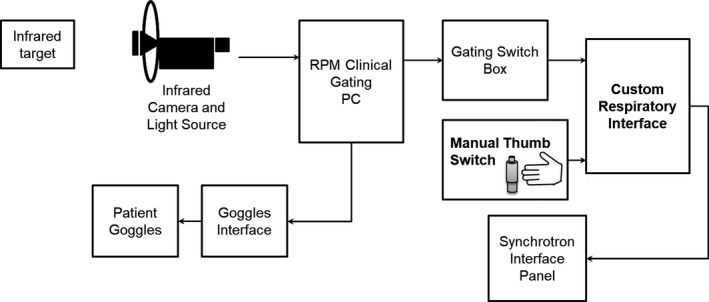
Respiratory gating interface system diagram. The interface provides health logic (i.e., ready) signals to the real‐time position management system (RPM) software via the Varian “Gating Switch Box,” and in turn, our interface forwards gating (beam‐on/beam‐off) logic signals it receives from the RPM (via the same Gating Switch Box) to the synchrotron delivery/control system.

Working in collaboration with Mayo Clinic's Division of Engineering, a custom “Respiratory Interface” was designed and fabricated to provide compatibility between the RPM (Varian Medical Systems, Inc.) and the Hitachi Synchrotron (Fig. 1).

As part of the implementation of our motion mitigation program, a separate “rescanning” machine based on the iso‐layered repainting (also known as a “Max‐MU” threshold approach) was commissioned in Eclipse. Max‐MU repainting was achieved by simply setting the maximal MU/spot in the planning system to a value smaller than the actual deliverable maximum MU, in our case MU_MAX_ = 0.005, (only 2.5× our minimum deliverable MU of 0.002).

#### 2.A.3. System performance

##### Acceptance testing

Our institution and the manufacturer agreed on a 200 and 0.5 ms proton beam delivery latency specification for beam‐on and beam‐off respectively. The acceptance testing performed involved generation of TTL gating logic signals with a vendor‐provided device, with simultaneous monitoring of these logic signals in relation to synchrotron delivery signals (e.g., gantry room bending magnet) during delivery of treatment plans sent via ARIA in clinical/DICOM mode (with a tag in the DICOM metadata indicating that the given plan requires an external gating signal).

##### Commissioning

For bench testing purposes, a plan was designed to deliver a simplified spot pattern to a one‐dimensional (1D) planar motion phantom. The phantom moved in a 1D pattern/amplitude proportional to a prerecorded patient RPM breathing trace. The plan targeted three imaging BBs (affixed to the anterior phantom slab surface prior to simulation CT scanning) using a single AP beam with three (BB) spot locations and six consecutive energy layers (treating same three spot locations on a beams‐eye view) with 1 MU per spot location, per layer (18 MU in total). The phantom was localized in the treatment room with a stereoscopic, kV x‐ray‐based clinical image‐guidance system (aligning to the BBs). The plan was delivered in three separate runs: (a) static (no motion); (b) free‐breathing (1D stage motion) with no gating; (c) gating (1D stage motion with duty cycle incorporating the 20%–60% phases of breathing, corresponding to expiration. A sheet of EBT3 film was placed on top of the phantom prior to each delivery to capture the delivered upstream/superficial spot/fluence pattern. The films were digitized (48 bit, three color channels) on a high‐resolution flatbed scanner and dose‐converted using FilmQA Pro [Ashland]).

### 2.B. Active motion management program

The following sections describe our clinical decision‐making process for patients undergoing proton treatment simulation with abdominal or thoracic moving tumors.

#### 2.B.1 Simulation procedure

Currently, all motion‐managed cases are simulated head‐first supine, with 1.5 mm CT slices. A 4D‐CT is required, and the data set is used for two purposes: as a tool for motion evaluation and to create a 4D‐average representation for treatment planning. Prior to 4D‐CT acquisition, the respiratory trace derived from the RPM (used to generate the 4D‐CT binning) is evaluated for reasonable regularity (regarding both cycle‐to‐cycle amplitude and period) and for having one discernable inspiratory “peak” per respiratory cycle. If an irregular trace is observed or if the motion analysis performed on the reconstructed phases yields displacements larger than 10 mm, a breath‐hold scan may be additionally performed.

Our institution's protocol for BH simulation is determining the BH gate level based on patient comfort (typically moderate‐deep inspiration — mDIBH; with an RPM amplitude gate width of 5 mm); then subsequently acquiring three BH scans to verify that BHs are performed consistently by the patient. The choice of which scan to use is oftentimes based on visual review of the fused BH CTs so as to pick the scan that best approximates the “mid‐position” anatomy. Sometimes we may objectively throw out one or more of the multiple BH scans from consideration because of poor compliance observed with respect to the target BH gate during the scan. (This is necessary because the CT scan is not truly “gated” like the proton therapy machine.) Deep‐inspiration BH is not used due to reproducibility concerns.^18^ A coaching video is initially shown to the patient with the goal of familiarizing the patient with the procedure and RPM's video goggles and visual interface.

The decision on whether to use Ph‐G and selection of the associated duty cycle is made by the physician in direct consultation with the medical physicist. This decision is ideally made immediately after simulation (i.e., prospectively) on the basis of target and upstream heterogeneity (i.e., diaphragm) motion amplitudes. In these cases, we typically select a duty cycle comprising 4D‐CT phases near end of exhale such that residual target/fiducial motion is below 1 cm. Otherwise, this decision is made retrospectively during treatment planning (when physician contours/target volumes are available) on the basis of both (preliminary) treatment plan robustness against the extreme 4D‐CT motion phases as well as consideration for potential dosimetric interplay. Particular attention is paid to (a) the plan evaluation at the respiratory extremes (0% and 50% 4D‐CT phases); (b) motion of the gross tumor volume (GTV) or clinical target volume; and (c) motion of contextual anatomy in the path of any given beam proximal to target (causing WET variations). If it is determined that there is significant loss of coverage on the extreme 4D‐CT motion phases which cannot be adequately addressed via more robust planning maneuvers (HU override strategies and/or beam‐specific spot‐scanning target volume — STV — margin adjustments), or that the motion of the GTV exceeds approximately 1 cm, gating is typically considered with preliminary duty cycle chosen in consideration of the above‐mentioned motion‐related cutoffs. This requires corresponding regeneration of internal target volumes (ITVs) and STVs, with plan reoptimization. Whether prospectively selected or not, the chosen extreme 4D‐CT motion phases are used for further plan robustness evaluations.

#### 2.B.2 Motion management guidelines and treatment planning

**Figure 2:**
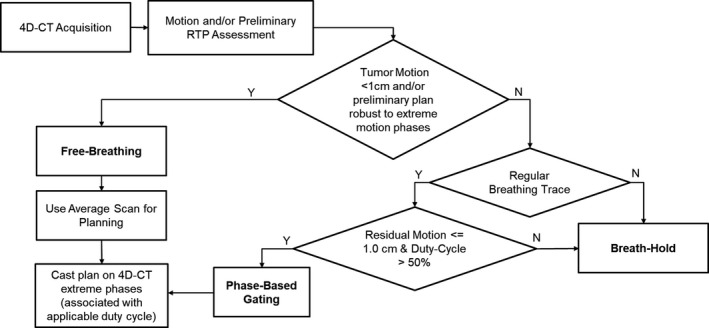
Simplified decision‐making scheme for patients treated with respiratory‐gated spot‐scanning proton therapy. In addition, repainted delivery is used in cases that have ≥5 mm residual motion and, typically these plans are created with 2–4 fields.

Our clinic's motion‐management generic decision‐making scheme is shown in Fig. 2. For BH cases, ITVs are typically constructed considering all acquired BH scenarios (treatment plans are typically also calculated on all BH scans to evaluate robustness to observed BH variation). SFO is preferred as the default SSPT optimization approach, whereas MFO is considered when normal tissue sparing must be improved based on physician's judgment. Free‐breathing plans (with and without gating) normally employ Max‐MU repainting as an interplay mitigation strategy when residual tumor motion, or WET variation, exceeds approximately 5 mm. Generally speaking, repainting is not deployed in combination with a breath‐held treatment.

##### Site‐Specific Details

Distal Esophageal disease is typically treated with two posterior left/right oblique fields with a hinge angle of approximately 40°. Isocenter is placed taking into account the geometry of our treatment rooms and the excursion range of the robotic couch. Unacceptable respiratory motion during free‐breathing treatment of esophageal cancer can be mitigated with either breath holding or phase gating, with preference for the latter due to robustness considerations given our clinical experience to date. WET variation due to diaphragmatic excursion is considered in addition to the standard criteria outlined in Fig. 2.

Hodgkin's disease is typically treated with two combinations of beam angles: two anterior obliques and a straight posterior or two posterior obliques with a straight anterior field. Unacceptable motion can be managed comfortably with either BH or Ph‐G. The criteria outlined in Fig. 2 are generally followed for this site with repainting employed when any portion of the target exhibits motion >5 mm.

Liver cases are typically treated with three fields on the right side. The angles selected must ensure minimal WET variation (e.g., when treating near the liver dome) while minimizing normal liver dose. When target motion is <1 cm and there is no overlap between ITV and stomach or bowel, the treatment is delivered free‐breathing in combination with repainting. If target motion is above this 1 cm threshold, breath‐holding (BH) may be used, although Ph‐G treatments are typically preferred when the tumor is adjacent to the liver dome.

Breast/Chest‐wall lesions are typically treated with two enface oblique fields. A 4.5 cm range shifter is used to allow superficial coverage. Similar to conventional x‐ray therapy, breath‐hold may be utilized to geometrically displace the heart away from the targeted chest wall volume. Generally, chest wall motion observed is <5 mm (and is often principally along the en‐face beam direction); hence, when breath holding is not used for heart sparing, free breathing treatments are preferred.

##### Deviations from standard practice

For patients with a highly irregular breathing trace and noncompliant for BH, coached shallow breathing may be considered as alternative. In shallow breathing, the patient is given feedback through the goggles to maintain a small‐amplitude breathing trace. A phased‐based 4D‐CT for residual motion assessment and planning can be acquired and reconstructed with the RPM used in an amplitude mode.

Another source of deviation from our motion management SOP's is the presence of metal hardware in the potential beam path. Geometric beam avoidance of these objects would be a logical preferred strategy, likely allowing for SFO planning. However, in some cases this compromise may excessively hamper the ability to spare critical structures. In these unique scenarios, MFO, combined with more elaborate STVs and robust optimization including inter‐field variability likely provides the best compromise.

#### 2.B.3 Image‐guided radiation therapy (IGRT) process

**Figure 3:**
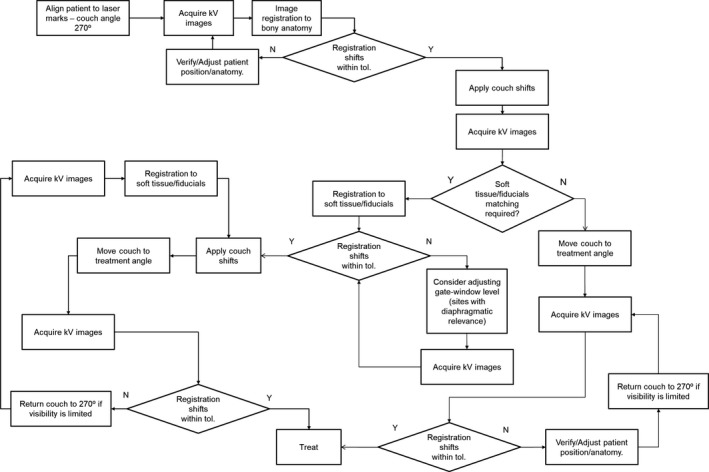
General decision‐making scheme for IGRT using our standard stereoscopic kV imaging system. X‐Ray imaging always starts at our defined “setup” couch angle (270°); verification x‐ray imaging is typically performed at the actual treated couch angles. Issues with localization encountered during treatment may require us to return to couch angle 270°.

Our standard IGRT methodology is summarized in Fig. 3 and is performed with an oblique stereoscopic kV imaging system and six degrees of freedom couch. Couch angle 270° provides a traditional geometry for stereoscopic kV imaging and images unobstructed by the couch robotics, and all treatments begin with this configuration. For mobile soft tissue based targets, we perform a 6D bony match (generally based on spine) and then make adjustments (translation only) to align to fiducials/clips (or target if visible). Performing three‐dimensional shifts off a nominal 6D bony registration may not always be advisable, depending on the beam arrangement, because of implications for OAR sparing or possible WET changes. As a consequence, for BH cases we may opt to adjust the breath‐hold gate level to allow both bony and soft‐tissue alignment to be consistent with simulation. After isocenter localization at couch angle 270°, verification x‐ray imaging may also be performed at the actual treatment couch angles. Optical surface imaging is increasingly utilized in our clinic, primarily as a tool for reduction in frequency of x‐ray imaging: e.g., monitoring accuracy of couch rotation, global patient motion, and BH variability.

#### 2.B.4 Treatment plan verification

Per standard clinical practice, motion‐managed treatment sites are, by default, subject to a “plan verification” regimen, requiring the patient's treatment plan to be recalculated on CT scans obtained weekly (using the given motion management technique, where applicable). Physicians review dose coverage anatomically, in some cases generating dose volume histograms for either rigidly or deformably propagated targets and OAR structures. Unacceptable target coverage and/or unintended and significant OAR overdosing will trigger the replanning process. In this case, the revised plan can improve in overall quality in that its robustness can be evaluated using both the verification CT(s) as well as the prior/initial planning CT.

### 2.C Retrospective patient data analysis

#### 2.C.1 Patient distribution

**Figure 4:**
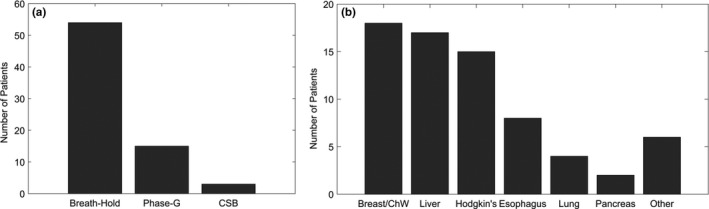
(a) Number of patients treated under three different motion management strategies involving the real‐time position management system, namely breath‐hold, phase‐gating, and coached shallow breathing. (b) Distribution of sites treated using these types of motion management.

More than 70 Patients with thoracic and abdominal lesions were treated with SSPT in combination with an active motion management strategy involving the RPM between October 2015 and January 2018. The frequency distribution of most commonly‐observed sites is shown in Fig. 4. Our institutional review board approved the usage of plan files for the current study.

#### 2.C.2 Efficiency analysis

Delivery time data were retrieved from our Record & Verify system for patients treated with BH ± repainting, Ph‐G ± repainting, and FB ± repainting. For all fractions of each patient, the time stamps of beam‐on and beam‐off signals were recorded and averaged. In addition, information regarding number of fields and energy layers used was incorporated in the analysis — in‐house C# code making use of the Eclipse Scripting API (Varian Medical Systems, Inc.) was developed to extract this information automatically. During pretreatment QA procedures, the included BH and Ph‐G plans were delivered in continuous mode; this baseline timing data was available from our patient‐specific QA treatment records.

## 3 RESULTS

### 3.A Commissioning test

**Figure 5:**
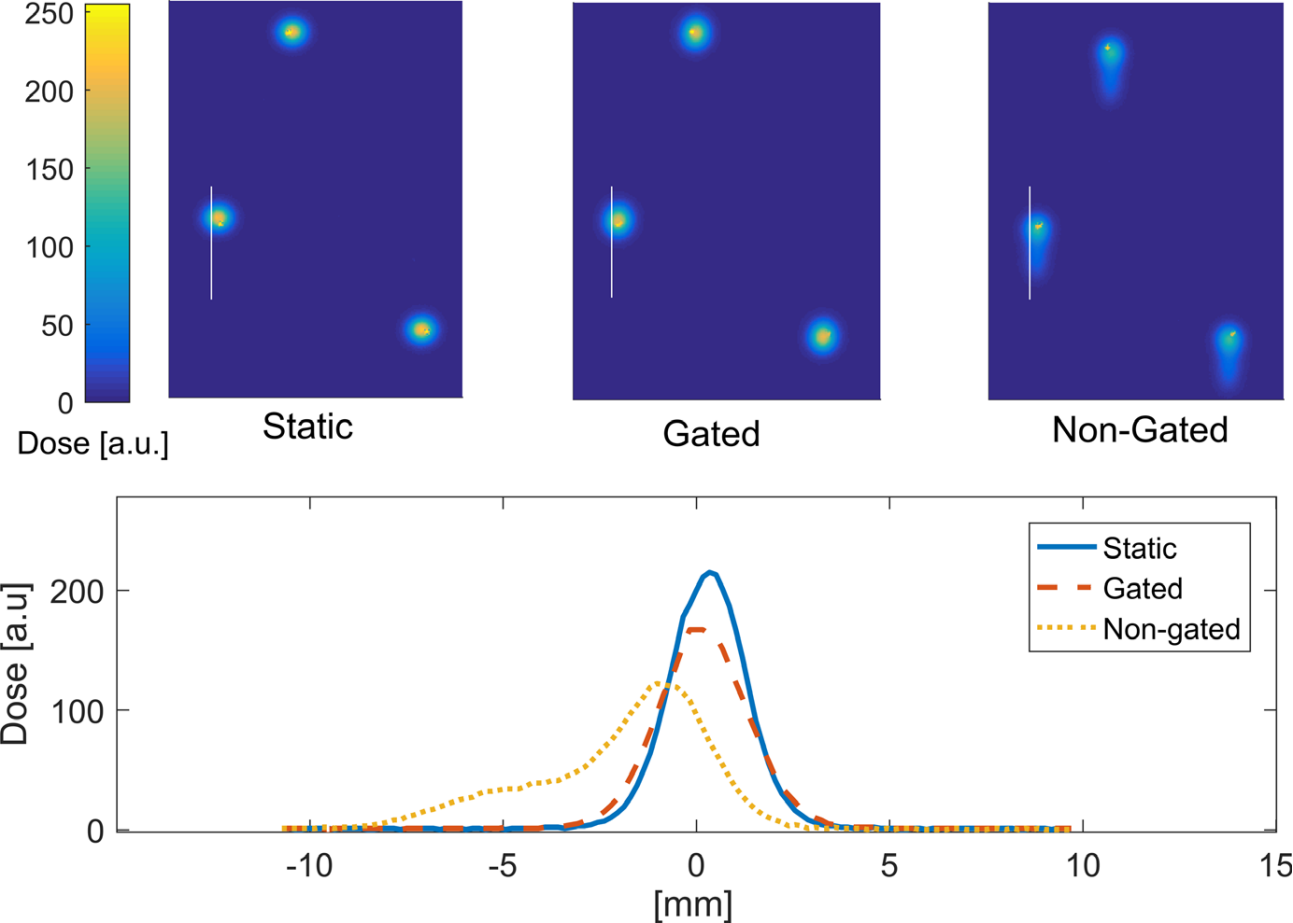
Film analysis of gated delivery test with a motion phantom. The position of the BBs (drawn “dots”) was used for alignment. The white line indicates the location where 1D profiles were extracted — to avoid interference with the drawn dots.

Figure 5 shows the dose‐converted EBT3 film planes for each of the three motion phantom deliveries (static, free‐breathing and gated). The free‐breathing ungated delivery spread the dose over a range of 8 mm, whereas the static and gated spots demonstrated a FWHM of 2.38 and 2.55 mm, respectively, validating the effectiveness of expiration‐gating for this simple plan. The results of the commissioning measurements give us confidence in the overall performance of the gating interface. The system is periodically monitored in our annual QA procedures.

**Figure 6:**
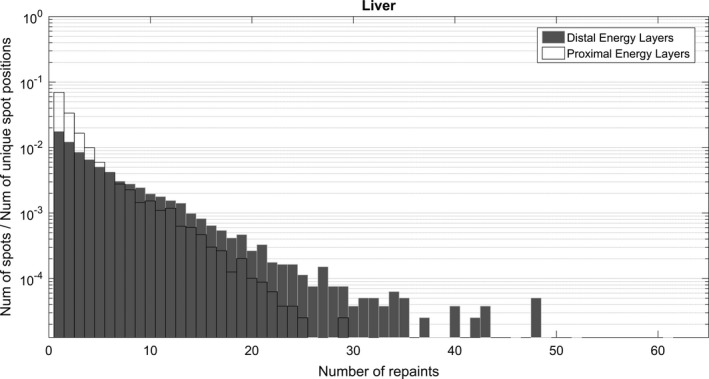
Number of repaints per spot normalized to the total number of unique spot positions. Energy layers were broken down in two groups — distal (1/3) and proximal (2/3). The histograms were created using DICOM RTPlan files of treatments were “Max‐MU” based rescanning was used.

Figure 6 shows spot repainting distribution/frequencies for the “repaint” machine of a hypo‐fractionated liver case with no range shifter, organized by energy layers utilized (collection of distal 1/3 vs proximal 2/3 of utilized layers). The histograms reveal two general features of Max‐MU‐based repainting: some spots are being repainted a large number of times (e.g., >30) and the average number of repaints is higher for distal layers (due to higher‐MU spots being utilized).

### 3.B Efficiency analysis

**Figure 7:**
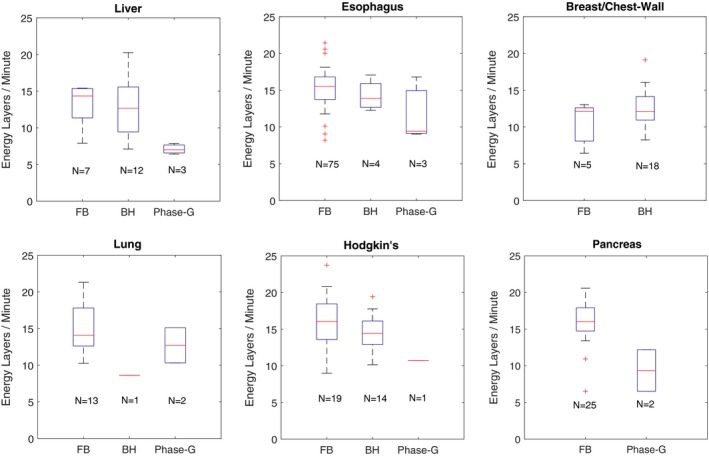
Delivery time data retrieved from our Record & Verify system for patients treated using some form of motion management. The box plots show a comparison of treatment efficiency for sites treated with three different modalities: free‐breathing + repainting (FB), breath‐hold (BH), and phase‐based gating. The boxplots present the data distribution as follows: maximum, 75% quartile, median, 25% quartile and minimum. Outliers are plotted as single markers.

The average delivery time per field for different sites is quantified in Fig. 7 using energy layers delivered per minute as a proxy for delivery efficiency. The plot compares treatments delivered using BH, Ph‐G, and FB + repainting. Due to relatively small sample sizes for these treatment sites and motion management techniques, no substratifications were included in terms of, for example, plan complexity or treatment volume. The general trend observed is that, compared with FB treatments (+repainting), Ph‐G treatments take longer to deliver than BH treatments. In the case of lung, the majority of patients included in the study were treated with Free‐Breathing (N = 13), and only a few of them included active motion management (N = 3). The inverted trend between BH and Ph‐G for this particular site can likely be attributed to patient‐specific characteristics such as, target size or patient breathing performance at the time of treatment. Which is to say, given the small numbers we were likely comparing dissimilar plans on average.

**Figure 8:**
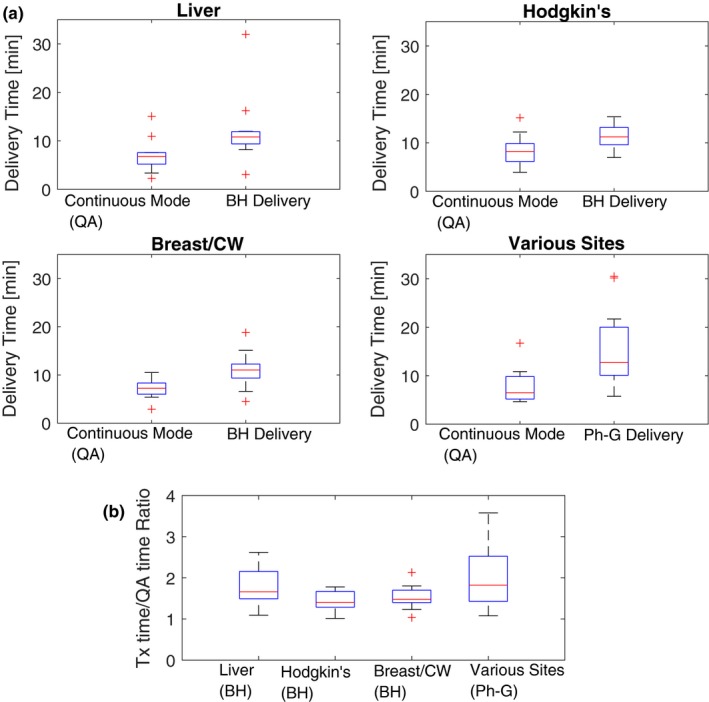
Comparison of treatment time and continuous delivery mode (QA) using boxplots as previously described (Fig. 7). The sites in the phase‐gated bin for various sites include: liver, esophagus, lung, pancreas, and bile‐duct. The results are presented in two formats: (a) contrasting absolute time of each delivery mode and (b) ratios between treatment time and continuous delivery.

Figure 8 compares the total beam delivery times of plans delivered using motion management strategies vs the same plans run in continuous mode (used for patient‐specific QA). On average, beam delivery time for BH treatments is extended from 6.59 to 9.33 min (+48%), whereas Ph‐G treatments are extended from 7.3 to 13.0 min (+82%). The time penalty will propagate to each patient's appointment time and consequently increase the wait time for other patients queued for the proton beam.

## 4 DISCUSSION

Implementing BH and Ph‐G treatments in our practice has come with an efficiency cost. Longer treatment times are observed throughout all the techniques implemented at our site including FB+repainting. Ph‐G cases suffer from the highest time penalty due primarily to two factors, namely (a) the addition of repainting to gated delivery: many of our Ph‐G plans also are combined with re‐painting when residual motion within the gate is deemed unacceptable; (b) the effective/realizable duty cycle is often less than the planned/ideal duty cycle, due to variable patient breathing as well as synchrotron control system behavior in the context of unpredictable beam‐on gating signals (since beam cannot be held infinitely due to space‐charge‐related instabilities). In our experience, the effective duty cycle of the system is significantly improved by adjusting RPM's “predictive filter.”^19^ As shown in Fig. 7, in some cases BH treatments can achieve similar delivery speed performance as FB + repainting; this is explained by the short beam‐on time per rescan produced by iso‐layered repainting.^20^ Other factors which extend treatment times are poor patient compliance and plan complexity. In the Breast/Chest‐Wall group of patients, BH seems to outperform FB + repainting, however, this apparent finding is explained by a bias in our data: the FB + repainting cases in this analysis are all hypo‐fractionated partial‐breast irradiation treatments whereas all of the BH cases received more conventional fractionation.

Long treatment times are undesirable not only due to the effect of reduced patient throughput, but most importantly because they will lead to patient discomfort and possible intrafractional patient movement. Machine performance or effective delivery speed plays an important role in the overall feasibility and efficiency of SSPT motion management. Multi‐Energy Extraction (MEE), an alternative proton beam extraction method that can reduce the energy‐switching time approximately tenfold, is now routinely used at our facility but was not available during the data collection period associated with this study. Preliminary results show that MEE may reduce our overall treatment delivery times by between 35%^21^ and 50%. Another element associated with delivery time, is the Bragg‐peak width of the available proton energies. The required number of energy layers can be significantly reduced using an energy smearing device such as a mini‐ridge filter (MRF). The usage of an MRF for range spreading in our Hitachi nozzle has been explored by Remmes et al.^22^ and Courneyea et al.^23^ We plan to deploy this device clinically at our facility very soon, recognizing its potential relevance in terms of treatment efficiency, motion management, and general plan robustness.

Current and future developments of our motion management program include the following: evaluating surface monitoring as a beam‐gating signal, exploring alternative rescanning techniques, and implementing practical 4D dosimetric verification tools. The evaluation of 4D robustness for esophageal plans was reported previously^24^; more recently, a generalized 4D plan calculator (including dynamic interplay effect) accumulator based on our GPU‐based Monte Carlo dose algorithm^25^ has recently become available for clinical evaluation.^26^ This 4D GPU Monte Carlo calculation framework is currently being incorporated into a 4D robust plan optimization framework.

Replanning frequency per treatment site is the subject of an ongoing internal investigation, which is outside the scope of this report. A preliminary and high‐level review of these data suggest that replanning is required for approximately 25% of cases involving disease sites relevant for motion management. However, since each patient, each treatment site and each specific motion management strategy pose unique challenges, more in‐depth study and granular reporting is needed.

## 5 CONCLUSIONS

This work presents a review of the current processes at our institution in the context of motion management. After developing and testing an in‐house hardware solution for motion management, we subsequently established what we view as conservative decision‐making criteria to ensure dosimetrically acceptable outcomes when dealing with moving targets. With respect to delivery efficiency, our findings have impacted our clinical practice by adding treatment time as a parameter of consideration when distinguishing between different motion‐mitigation strategies. The methodologies and decision‐making processes presented here are relevant for other proton or particle therapy centers that are considering implementing active motion management.

## CONFLICT OF INTEREST

The authors have no conflicts of interest to disclose. No external funding was received.
